# Editorial and Miscellaneous

**Published:** 1864-05

**Authors:** 


C. S. Medical & Surgical Journal.

RICHMOND, MAY, 1864.

*AYRES & WADE......PUBLISHERS AND PROPRIETORS.*

### *Amputation, Disarticulation and Resection Statistics of the Confederate States Army.*

We are indebted to the Surgeon-General for the privilege of preparing from the original papers now on file in his office, the following operative statistics, as reported by officers of the medical staff, from the beginning of the war down to the 1st of February of this year:

Amputations of the Thigh, whole number, 507: Primary, 345; recovered, 213; died, 132; 38 per cent. Secondary, 162; recovered, 43; died, 119; 73 per cent.

Amputations of the Leg, whole number 464: Primary, 314; recovered, 219; died, 95; 30 per cent. Secondary, 150; recovered, 76; died 74; 49 per cent.

Amputations of the Arm, whole number 434; Primary, 294; recovered, 252; died, 42; 14 per cent. Secondary, 140; recovered, 87; died, 53; 37 per cent.

Amputations of the Fore-Arm, whole number 114: Primary, 69; recovered, 61; died, 8; 12 per cent. Secondary, 45; recovered, 35; died, 10; 22 per cent.

Disarticulations, whole number 135; Primary, shoulder joint, 79; recovered, 54; died, 25—31 per cent. Primary, elbow-joint, 4; recovered, 3; died, 1—Primary, wrist-joint, 7; recovered, 5; died, 2—Primary, hip-joint, 3; recovered, 1; died, 2—Primary, knee-joint, 5; recovered, 2; died, 3. Secondary, shoulder-joint, 28; recovered, 8; died, 20—71 per cent. Secondary, elbow-joint, 3; recovered, 2; died, 1—Secondary, knee-joint, 6; died, 6.

Resections, whole number 130: Primary, shoulder-joint, 41; recovered, 28; died, 13—27 per cent. Primary, elbow-joint, 25; recovered, 22; died, 3—Primary, wrist-joint, 2; recovered, 2—Primary, knee-joint, 2; died, 2. Secondary, shoulder joint, 26; recovered, 19; died, 7—21 per cent. Secondary, elbow-joint, 29; recovered, 23; died, 6—Secondary, wrist-joint, 1; recovered, 1—Secondary, hip-joint, 2; recovered, 1; died, 1—Secondary, knee-joint, 2; recovered, 1; died, 1.

Amputations of the Foot: Primary—Chopart's, 16; recovered, 13; died, 3—Symes's, 2; recovered, 2—Pirogoff's, 4; recovered, 2; died, 2. Secondary—Chopart's, 8; recovered, 7; died, 1; Symes's, 4; recovered, 4; (1 unsuccessful, requiring subsequent amputation above the ankle-joint.)

A vast number of additional operations are received, but without positive results, and, therefore, they have not been included in the above list.

We may be well satisfied with the results of these statistics, which, carefully excluding all doubtful cases, are compiled from those operations only that have reached a positive conclusion. A general summary of the above tables shows that the mortality after 1,814 operations, including amputations, resections and disarticulations, amounted to 632, giving a death ratio of 34 per cent.

By referring to the mortality tables after amputations subsequent to many of the great battles of modern days, taken from the pages of Lagouest, which will be found in our Chronicle for this number, the reader will be able to draw his own comparisons.

The only statistics on this subject from the Federal army we find in the United States Army and Navy Journal for November, 1863, which gives the amputation statistics for September, October, November and December of 1862, as follows: Whole number, 1,342; deducting 516 under treatment January 1, 1863=826. Of this number, 336 died; a mortality of 40 per cent.

The journal to which we owe the above observation gives the following table: Whole number, 1,342; returned to duty, 100; furloughed, 25; deserted! 11; discharged, 350; died, 336; secondary operation, 34; under treatment, January 1, 1863, 516.

### *The Prospect before Us.*

It is gratifying to inform our readers that this Journal, in spite of the many difficulties which have surrounded its rise and progress, has already attained a larger circulation than was ever reached before by any Southern medical periodical, and promises, in the future, to surpass the most sanguine expectations of its friends.

The usual impediments to the success of similar enterprises, it is true, have been fortunately spared us. Subscriptions have flowed in rapidly, and, more important still, contributions of a useful and instructive character, and in great variety, reach us from every source. Our communications with the literature of other countries have been singularly successful, for we pen our hasty editorial with the *April* number of the London "Lancet" lying on the table.

The great difficulty, not yet overcome, is to be found in the want of the various elements necessary to the mechanical preparation of the Journal, more particularly the want of paper, although other minor troubles might be mentioned. We need room to do adequate justice to the wealth of material, both original and selective, now in hand, and our monthly installments are felt by all to be condensed to a fault, and requiring only more space to add vastly to their interest and value.

We may venture to hope that this objection will shortly be obviated, by enlarging our publication to twice its present size, and in the meantime we must ask our many friends and contributors to exercise that patience and forbearance which may be reasonably claimed under the circumstances.

To obviate the delay in receiving the Journal, so much complained of by the army subscribers, the publishers have made arrangements to pre-pay their postage at this office, which will prevent a recurrence of these repeated disappointments.

Let us hope that, by the commencement of our *second* half year, with increased size and increasing aid from our collaborators, and with an earnest desire to do justice to those who have promptly and generously given us their support, we may not fail in winning from our readers a moderate amount of approbation and confidence.

